# Impact of lunch provision on anthropometry, hemoglobin, and micronutrient status of female Cambodian garment workers: exploratory randomized controlled trial

**DOI:** 10.1186/s40795-019-0297-4

**Published:** 2019-07-08

**Authors:** Jan Makurat, Natalie Becker, Frank T. Wieringa, Chhoun Chamnan, Michael B. Krawinkel

**Affiliations:** 1 Institute of Nutritional Sciences, Justus Liebig University Giessen, Wilhelmstrasse 20, 35392 Giessen, Germany; 2 UMR 204 Nutripass, Institut de Recherche pour le Développement (IRD), IRD / UM / SupAgro, 911 Avenue d’ Agropolis, 34394 Montpellier, France; 3 Department of Fisheries Post-Harvest Technologies and Quality Control (DFPTQ), Fisheries Administration, Ministry of Agriculture, Forestry, and Fisheries, 186 Preah Norodom Boulevard, 12000 Phnom Penh, Cambodia

**Keywords:** Lunch provision, Staff canteen, Garment factory, Cambodia, Randomized controlled trial, Malnutrition, Underweight, Anemia, Micronutrient deficiency, Industrial worker

## Abstract

**Background:**

Lunch provision is expected to improve the nutritional status of Cambodian garment workers. The objective of this study is to evaluate the effects of a model lunch provision through a canteen on anthropometry, hemoglobin, and micronutrient status in female garment workers in Cambodia.

**Methods:**

This exploratory randomized controlled trial was implemented at a garment factory in Phnom Penh, Cambodia. Female workers (nulliparous, non-pregnant) were recruited and randomly allocated into an intervention arm (workday’s lunch provision) and a control arm. Served lunch sets (~ 700 kcal on average) included diverse local dishes. Anthropometry (body mass index, weight, triceps skinfold thickness, and mid-upper arm muscle circumference), as well as hemoglobin, serum ferritin and soluble transferrin receptor, serum retinol binding protein, and serum folate concentrations were assessed at baseline and after 5 months of lunch provision. A general linear model with adjustments for baseline values was used to estimate intervention effects for each outcome variable.

**Results:**

Two hundred twenty-three women were recruited (*n* = 112 control and *n* = 111 intervention). 172 (*n* = 86 in each arm) completed the study. Baseline prevalence of underweight, anemia, depleted iron stores, and marginal iron stores, were 31, 24, 21, and 50%, respectively. Subjects were not affected by frank vitamin A or folate deficiency, whereas 30% showed a marginal folate status. Overall, mean changes in anthropometric variables, hemoglobin, and retinol binding protein were marginal and not significant among intervention subjects. Mean folate concentration increased insignificantly by + 1.1 ng/mL (− 0.02, 2.2) (*p* = 0.054). On the other hand, mean ferritin decreased by − 6.6 μg/L (− 11.9, − 1.3) (*p* = 0.015). Subgroup analysis prompts that effects are differently pronounced according to the baseline status of workers.

**Conclusions:**

Findings indicate that model lunch sets provided a beneficial amount of dietary folate, but need to be revisited for iron content and/or iron bioavailability. It is believed that distinct positive effects on anthropometry, hemoglobin, and micronutrient status can solely be expected in malnourished individuals. The authors suggest that similar larger trials, which include sets adapted to the concrete needs of workers affected by underweight, anemia and/or definite micronutrient deficiencies, should be performed.

**Trial registration:**

The trial was registered at the German Clinical Trials Register (9 January 2015, Identifier: DRKS00007666).

## Background

More than 600 export-oriented garment factories operate in Cambodia, which demonstrates the key role that this industry plays in the country’s economy [1]. Almost 90% of their 600,000 employees are female, mainly young women who migrate from disadvantaged low-income rural households [2–4]. Located in and around Phnom Penh, the capital of Cambodia, factories are usually owned by foreign investors [1, 5, 6]. They implement low value-added activities and rely on inexpensive labor [5, 6]. The 2017 minimum wage for Cambodian garment workers amounted to 153 USD/month, which was notably lower in previous years [1]. This fact inevitably drives workers to depend on bonuses and overtime work [3, 7, 8], and yet more than 50% of the total salary is budgeted to support family members [3, 4, 7, 8].

Socio-economic surveys have concluded that an appropriate diet is likely to be out of reach [3, 7, 8]. It is reported that workers spend on average ~ 1.5 USD/day on food, primarily at food stalls close to the factories, and that saving measures involve skipping of meals [3, 7, 8]. Despite the importance of the topic, sound data regarding the dietary intake of Cambodian garment workers are missing. Based on a small cross-sectional survey, NGOs reported a prevalence of 36% underweight among female workers [3]. On the other hand, a study conducted by the ILO in several factories found 14% of workers to be underweight and 45% to be anemic [9]. Finally, the authors reported on 31% underweight, 27% anemia, and a high prevalence of poor iron status (data from the present factory-based study) [4].

Malnutrition of women in reproductive age (with respect to underweight, anemia, and micronutrient deficiencies) is one of the great threats to public health in the country [10]. For instance, during gestation, malnutrition is linked to increased maternal morbidity and mortality, fetal and neonatal deaths, premature delivery, and low birth weight [11–13]. Anemia of nutritional origin is caused by poor diets that lack adequate amounts of iron, vitamin A (VitA), vitamin B12 (VitB12), and/or folate [11, 14]. In contrast, non-nutritional factors are especially hemoglobinopathies, menstrual blood loss, and parasite infestation [11, 15, 16].

The implementation of canteens serving lunch in Cambodian garment factories has been proposed as an adequate intervention to improve the nutritional situation of workers, to reduce morbidity and absenteeism, and thereby to increase productivity [17]. Nevertheless, convincing trials that verify these hypotheses are rare and the vast majority of factories does not hold a canteen, with the operation costs being the most critical factor for factory owners [17]. Despite the implementation of a first on-topic survey by the ILO [9], detailed insights are missing concerning the consequences of lunch provision on the nutritional and health status of Cambodian garment workers. Yet, this knowledge is essential to make informed choices on the setup and operation of staff canteens.

The current essay reports on the main outcomes of the LUPROGAR study (Lunch Provision in Garment Factories), a factory-based exploratory randomized controlled trial, whose objective is to determine the effect of a low-price model lunch provision via a canteen during workdays on anthropometry, as well as on the hemoglobin (Hb) and micronutrient status of female garment workers in Cambodia. Prior to this paper, the authors provided detailed information on the participants’ nutritional and health status at baseline [4], the low-price model lunch provision approach within this trial [18], and on the food consumption of study participants with and without access to the model lunch provision [19].

## Methods

### Study design and setting

LUPROGAR was a factory-based exploratory randomized controlled trial (two-group, 1:1 ratio, parallel), planned for a six-month period. The study was realized during 2015 at Apsara Garment Co. Ltd., an export-oriented garment factory in the commune Chom Chau in Phnom Penh. The vast majority of the 1300 employees were young women from low-income rural households. Working conditions, including six workdays per week, were assumed to match the overall conditions of employment in this industry. Apsara Garment Co. Ltd. was selected purposely, since the factory management showed interest in cooperating in this project.

### Participants

The study population included young non-pregnant nulliparous women employed by Apsara Garment Co. Ltd., intended to match the characteristics of the majority of Cambodian garment workers. To be eligible, women had to meet the inclusion criteria and provide written informed consent before enrollment. The inclusion criteria were: being nulliparous (to rule out any confounding from breastfeeding and/or recent pregnancy), non-pregnant, apparently healthy, and < 31 years old at the date of enrollment. The criteria for exclusion were: acute or chronic disease requiring treatment and/or medication (including Hb concentration < 7.0 g/dL and clinical signs of VitA- or iodine deficiency), handicaps impairing nutritional and/or health status, and employment as supervisor. Subjects excluded due to any health problem were referred for treatment.

Beginning of March 2015, the factory management and union representatives were briefed about the LUPROGAR trial. Following this, the study was declared during a meeting to all workers. Trained assistants obtained written informed consents at an information desk at lunch breaks and at the end of working days (mid of March till beginning of April 2015). Women were then invited to the enrollment and baseline assessment which was performed during working hours, including a clinical check-up by trained nurses (end of April 2015).

### Intervention

A temporary canteen was installed in a roofed outdoor area at the factory site [18]. Apsara Garment Co. Ltd. had never operated a staff canteen before. Within the LUPROGAR trial, it was envisaged to serve adequate full lunch sets (consisting of a stir-fried dish, a soup, a side item (cooked rice), and a fruit dessert) at reasonable costs (~ 1 USD/person/day) in collaboration with Hagar Catering and Facilities Management Ltd., an established Phnom Penh-based canteen service provider. Sets should provide about one third of the recommended dietary allowance (RDA) for non-pregnant women aged 19–30 years old (total ~ 700 kcal) [20]. Based on these standards, a biweekly menu (of 12 model lunch sets) was outlined in consultation with the caterer [18]. Focus was laid on accepted Cambodian dishes, using local foods and ensuring variety by providing cereals, various vegetables, animal source foods (meat or fish), and fresh fruits on a daily basis.

Following the enrollment and baseline assessment at the end of April 2015, daily free lunch provision on workdays for the intervention group was carried out by the caterer for 6 months from beginning of May until end of October 2015. Dishes were prepared according to consistent recipes at a professional kitchen located in Phnom Penh’s city center and delivered within 1 h. Stir-fried and soup dishes were reheated just before serving and the canteen staff was instructed to serve constant portion sizes. At the canteen, participants had free access to drinking water and locally used condiments (non-fortified soy/fish sauce and fresh red chili). After 1 month, the initial menu was slightly adjusted according to preferences expressed by workers via a short preference questionnaire. Access to the canteen was voluntary and recorded daily by an assistant. Table 1 presents the estimated nutritive value of the lunch sets. Further information on exact costs, components and ingredients, serving sizes, and corresponding nutritive value of single lunch sets can be found elsewhere [18].

**Table 1:** Estimated nutritive value of the low-price model lunch sets provided to female garment workers at a factory in Phnom Penh, Cambodia^a^

Nutritive value^b^	Mean	Min.	Max.
Energy, kcal (% of RDA)	697 (33)	591 (28)	793 (38)
Carbohydrates, g (% of RDA)	107 (37)	100 (34)	123 (42)
Protein, g (% of RDA)	23 (46)	16 (32)	30 (60)
Fat, g (% of RDA)	18 (34)	12 (23)	24 (45)
Dietary fiber, g (% of AI)	8 (32)	6 (24)	12 (48)
Vitamin C, mg (% of RDA)	111 (159)	24 (34)	212 (303)
Iron, mg (% of RDA)	6 (20)	4 (14)	12 (41)
Vitamin A, μg RAE (% of RDA)	331 (66)	61 (12)	799 (160)
Folate, μg (% of RDA)	175 (44)	29 (7)	477 (120)
Vitamin B12, μg (% of RDA)	0.7 (29)	0.2 (8)	1.5 (63)

^a^Among 12 various lunch sets (composed of stir-fry, soup, side item (cooked rice), and fruit dessert) provided over a biweekly rotating cycle (one set per day, at six workdays per week). Data from a preceding publication including detailed information on the biweekly menu, costs, ingredients, serving sizes, and estimated individual nutritive values [18]

^b^Following recommendations for non-pregnant women aged 19–30 years old from various sources: energy, protein (adjusted for 80% protein quality), vitamin C, iron (adjusted for 10% bioavailability), vitamin A, and folate [20]; carbohydrates [21]; fat and vitamin B12 [22]; and dietary fiber [23]

*Min* Minimum, *Max* Maximum, *kcal* Kilocalories, *RDA* Recommended dietary allowance, *AI* Adequate intake, *RAE* Retinol activity equivalent

### Outcomes

Given the exploratory trial design, the outcomes were planned as changes in body mass index (BMI, kg/m^2^), weight (kg), triceps skinfold thickness (TSF, mm), and mid upper-arm muscle circumference (MUAMC, cm) (as anthropometric variables), as well as changes in Hb (g/dL) and serum ferritin (FER, μg/L, inflammation-adjusted), soluble transferrin receptor (sTfR, mg/L), retinol binding protein (RBP, μmol/L, inflammation-adjusted), folate (ng/mL), and VitB12 concentrations (pmol/L) (as Hb and micronutrient status) of participants at follow-up (planned at 6 months).

### Data collection

Details regarding the questionnaires, anthropometric measurements, and the blood sample collection and analysis can be found in a previous publication [4]. In brief, trained assistants applied a pre-tested socio-economic status questionnaire at baseline. In the context of the clinical screening, trained nurses administered a pre-tested health questionnaire (baseline and follow-up). Weight, height, TSF, and mid-upper arm circumference (MUAC) were assessed by two trained assistants following CDC guidelines [24] (baseline and follow-up). All devices and measurement procedures were pre-tested under field conditions. Weight was measured to the nearest 0.1 kg, height to the nearest 0.1 cm, TSF to the nearest 0.2 mm (using a Tanner/Whitehouse caliper (Holtain Ltd., UK)), and MUAC to the nearest 0.1 cm. All measurements were taken twice and the mean was used for further analysis. Subjects were classified using defined BMI cut-off points [24]. Within normal weight subjects, a BMI between 18.5 and 20.0 kg/m^2^ was also designated as “low-normal BMI” [25]. MUAMC was calculated using the following equation [26]:

$$ \mathrm{MUAMC}=\mathrm{MUAC}-\left(\uppi\ \mathrm{x}\ \mathrm{TSF}\right) $$

Samples of non-fasting venous blood were taken by trained nurses (baseline and follow-up). Blood drops were put on a glass slide for subsequent twofold blood Hb measurement using a HemoCue Hb 301 photometer (HemoCue AB, Sweden). The mean was used in further analysis. Blood left in the syringe was then processed to obtain serum aliquots, which were kept frozen at − 25 °C [4] . Serum VitB12 was measured by electrochemiluminescence at the Pasteur Institute Cambodia (Phnom Penh, Cambodia), using a COBAS e 411 immunoassay analyzer (Roche Diagnostics, Switzerland). When analyzing follow-up subsamples, VitB12 results of controls and samples unexpectedly fell out of the certified ranges. Therefore, only baseline results on VitB12 are shown here. Remaining aliquots were shipped on dry ice to the Institute of Nutritional Sciences at the Justus Liebig University Giessen (Germany) and stored at − 25 °C until they were processed at the VitMin laboratory (Willstaett, Germany). FER, sTfR, RBP, C-reactive protein (CRP, mg/L), and α1-acid-glycoprotein (AGP, g/L) were determined by a sandwich enzyme-linked immunosorbent assay (ELISA) technique [27]. Serum folate was measured via a microbiological assay by using chloramphenicol-resistant *Lactobacillus rhamnosus* [28]. Both methods used pooled samples for quality control and certified samples (CDC, USA and Bio-Rad, USA) to establish calibration curves for each indicator. All values represent the mean of an independent double measurement. For folate, the maximum tolerated difference between duplicate measurements was +/− 40%, otherwise the result was not included in further analysis.

Anemia was defined according to established cut-offs [11]. Subclinical inflammation was defined as increased CRP (> 5 mg/L) and/or increased AGP concentrations (> 1 g/L), and categorized into three stages [29]. FER concentration was adjusted for inflammation by correction factors for each inflammation stage [29]. Iron deficiency was defined by depleted iron stores (adjusted serum FER < 15 μg/L) [11], marginal iron stores by adjusted serum FER ≥15 and < 50 μg/L [30], tissue iron deficiency by high serum sTfR (> 8.3 mg/L) [31], and iron deficiency anemia by Hb < 12.0 g/dL and simultaneous adjusted serum FER < 15 μg/L [11]. Serum RBP concentrations were used as a surrogate measure for circulating retinol to evaluate VitA status [32]. RBP values were likewise adjusted for the presence of inflammation by correction factors for each stage of inflammation [33]. VitA deficiency was defined by adjusted serum RBP < 0.70 μmol/L and marginal VitA deficiency by adjusted serum RBP values ≥0.70 and < 1.05 μmol/L [32, 34]. Folate deficiency was defined by serum folate < 3 ng/mL and marginal deficiency by serum folate ≥3 and < 6 ng/mL [35]. VitB12 deficiency was defined as serum VitB12 < 148 pmol/L and a marginal VitB12 deficiency as serum VitB12 ≥ 148 and < 222 pmol/L [36].

### Sample size

An explorative strategy was used to determine an appropriate sample size, since both, data on the nutritional status of Cambodian garment workers and data on the effects of lunch provision in this context were largely missing at time of trial implementation. G*Power (v.3.1.9.2, University of Kiel, Germany) was used to carry out the calculation. At a two-tailed 5% level of significance (alpha = 0.05) and a statistical power of 80% (beta = 0.20) to detect a small to medium standardized effect size of 0.35 (Cohen’s *d*) between both arms [37], 130 subjects in each group were required at follow-up. Considering a 20% loss to follow up, it was aimed at recruiting a total of 330 participants (165 subjects in each arm).

### Randomization

Simple randomization with a 1:1 ratio into an intervention arm (access to six-month lunch provision through local canteen during workdays) and a control arm (equal monetary compensation at the end of the trial) was conducted via assigning a computer-generated random number for each subject (identifying the allocation to intervention or control) by making use of the random number generator within SPSS (v.22.0.0.1, IBM Corp., USA) (prepared by study coordinator). Enrolled participants were individually assigned to groups by an assistant who was neither involved in the enrollment procedure nor in the assessments.

### Statistical analysis

Data of questionnaires and anthropometry sheets were double-entered by trained assistants using EpiData (v.3.1, EpiData Association, Denmark). Overall data management and statistical analyses were executed using SPSS (v.22.0.0.1, IBM Corp., USA). Evaluation only included participants who completed the follow-up, regardless of the actual individual adherence of intervention subjects to daily eating lunch at the staff canteen. Detailed baseline findings among all originally enrolled subjects can be found in a previously published paper [4]. A wealth index was computed to assess the socio-economic status of subjects’ households using principal component analysis [38]. The index was based on the following variables: number of rooms per household, people per room, main place of cooking, main type of fuel, main material of the floor, and ownership of a bank account, latrine, electricity, and several household assets (radio, television, non-mobile telephone, wardrobe, sewing machine, DVD player, generator, watch, motorcycle, motorcycle cart, car, and boat). Baseline background characteristics of the groups were summarized by using descriptive statistics.

In the primary analysis, a general linear model with adjustments for baseline values (covariates) was used to calculate marginal means per group with 95% CIs for each outcome variable at follow-up, as well as to estimate the intervention effects as marginal mean differences with 95% CIs and corresponding effect sizes (Cohen’s *d*). The significance was set at 5% (*p*-value < 0.05). In a secondary analysis, the same model was used to compute marginal mean changes with 95% CIs per group for each outcome variable within following subsets (based on the assumption that changes differ according to baseline status): for anthropometric variables, subgroups were underweight, low-normal BMI, and BMI ≥20.0 kg/m^2^ at baseline; for Hb, subgroups were moderate anemia, mild anemia, and not anemic at baseline; for FER and sTfR, subgroups were iron deficiency, marginal iron stores, and sufficient iron stores at baseline; for RBP, subgroups were marginal VitA deficiency and no VitA deficiency at baseline; and for folate, subgroups were marginal folate deficiency and no folate deficiency at baseline. Given the small sample sizes within subgroups, this secondary analysis was not suited to test for powerful statistical significance.

### Changes to procedure

Due to a relatively high number of participants who ceased to work and left the factory (primarily as a result of a change of the main purchaser and a great part of management), the follow-up was antedated by 1 month in order to minimize the number of further dropouts. Hence, the endline assessment was conducted after 5 months (beginning of October 2015) instead of after 6 months. The canteen operated as planned until end of October 2015.

## Results

### Baseline characteristics

**Fig. 1:**
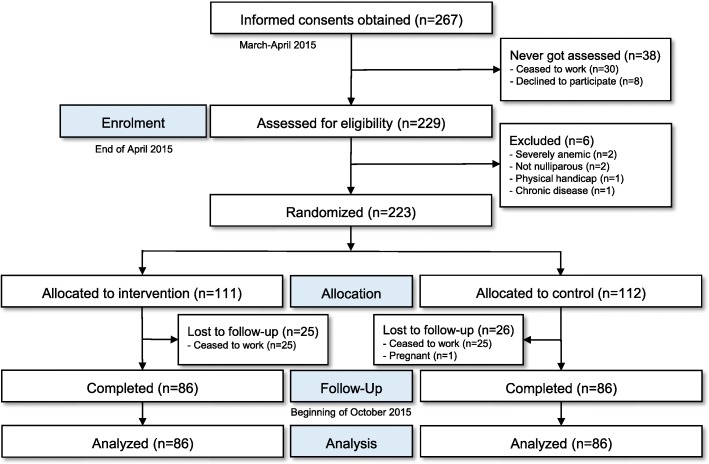
CONSORT flow diagram of the trial. The intervention group had access to free lunch provision on workdays through a canteen for 5 months.

Between 14 March and 4 April 2015, a total of 267 female workers signed the informed consent prior to enrollment (Fig. 1). At the enrollment procedure, which took place from 21 to 29 April 2015, 229 workers were present whereas 38 were not (*n* = 30 ceased to work and *n* = 8 refused to participate). Further six workers were excluded from participation at the clinical screening (*n* = 2 with Hb < 7.0 g/dL, *n* = 2 not nulliparous, *n* = 1 with physical handicap, and *n* = 1 with chronic disease). The remaining 223 women were randomly assigned and access to free lunch provision for the intervention group started in early May 2015.

The follow-up assessment took place from 1 to 10 October 2015. One hundred seventy-two women (77%) completed the follow-up (*n* = 50 ceased to work and *n* = 1 became pregnant), with endline data available for anthropometry. Dropouts were equally distributed across groups. The count of incomplete blood values for both time points (due to refused blood sampling, missing aliquot, or deviating duplicate measurement) was low for Hb (*n* = 2 of 172), FER, sTfR, RBP, CRP, and AGP (all *n* = 4 of 172), but slightly higher for folate (*n* = 21 of 172). Follow-up values for VitB12 were not available as described in the Methods section. Within the actual intervention period of 5 months, intervention subjects on average (mean) visited the staff canteen on 85% of total days (median was 92%).

Overall, participants had a mean ± SD age of 21 ± 3 years and a total monthly salary of 195 ± 34 USD. Of the total, 94% were single (*n* = 162 of 172), 67% stayed at a nearby shared room for rent (*n* = 115 of 172), and 63% (*n* = 109 of 172) worked as sewer. 63% (*n* = 108 of 172) reported a preceding employment in another garment factory. Baseline equivalence in background characteristics amongst groups was given (Table 2), despite a 23% dropout.

**Table 2:** Baseline background characteristics of female Cambodian garment workers with completed follow-up by group^a^

	Group	
	Intervention	Control
Total, *n* (%)	86 (50)	86 (50)
Age, years	21 ± 3^b^	21 ± 3
Weight, kg	46 ± 6	47 ± 6
Height, cm	153 ± 5	154 ± 6
School attendance, years	7 ± 2	7 ± 2
Marital status, *n* (%)		
Single	81 (94)	81 (94)
Married	4 (5)	4 (5)
Widowed	1 (1)	1 (1)
Hometown province, *n* (%)		
Phnom Penh	2 (2)	5 (6)
Others	84 (98)	81 (94)
Duration of employment in factory, months	14 ± 9	13 ± 9
Monthly basic salary, USD	131 ± 14	131 ± 9
Last monthly salary (incl. bonus, overtime, and allowance), USD	198 ± 37	191 ± 31^c^
Job type in factory, *n* (%)		
Sewing	52 (60)	57 (66)
Quality control	16 (19)	12 (14)
Buttoning	8 (9)	4 (5)
Cutting	4 (5)	3 (3)
Packaging	3 (3)	3 (3)
Others	3 (3)	7 (8)
Accommodation on workdays, *n* (%)		
Hometown, family household	18 (21)	32 (37)
Nearby place of friend/family	4 (5)	1 (1)
Nearby shared room for rent	63 (73)	52 (60)
Nearby private room for rent	1 (1)	1 (1)
Number of people in family household	4.6 ± 1.4	5.2 ± 1.6
Wealth index of family household	1.9 ± 2.8	2.4 ± 3.6
Participant’s monthly payment to family household, USD	119 ± 39	122 ± 41^d^
Family household’s primary source of income, *n* (%)		
Wage employment	46 (53)	54 (63)
Farming	22 (26)	17 (20)
Casual labor	6 (7)	10 (12)
Business/petty trade	7 (8)	4 (5)
Others	5 (6)	1 (1)

*USD* United States Dollar

^a^Total *n* = 172

^b^Mean ± SD (all such values)

^c^*n* = 82 (*n* = 4 newcomer (≤1 month of employment) without previous monthly salary from this factory)

^d^*n* = 85 (*n* = 1 participant without monthly payment to family household)

Data on the nutritional status and prevalence rates of anemia and micronutrient deficiencies are summarized in Table 3. At baseline, the prevalence of subclinical inflammation was 1% (*n* = 1 of 168 (*n* = 1 control)) for incubation (CRP > 5 mg/L only), 1% (*n* = 1 of 168 (*n* = 1 control)) for early convalescence (AGP > 1 g/L and CRP > 5 mg/L), and 7% (*n* = 12 of 168 (*n* = 4 intervention and *n* = 8 control)) for late convalescence (AGP > 1 g/L only). At 5 months, the prevalence was 1% for incubation (*n* = 1 of 171 (*n* = 1 control)), 1% for early convalescence (*n* = 2 of 171 (*n* = 2 control)), and 6% (*n* = 11 of 171 (*n* = 7 intervention and *n* = 4 control)) for late convalescence. Mean values at baseline for outcome measures are included in Tables 4 and 5. No significant differences between groups were noticed for anthropometric variables and concentrations of Hb, FER, sTfR, and folate. Although, mean RBP concentration was somewhat higher among control subjects (1.49 ± 0.31 vs. 1.37 ± 0.26 μmol/L).

**Table 3:** Nutritional status, anemia, and micronutrient deficiencies at baseline and 5 months (follow-up) in female Cambodian garment workers by group^a^

	Group	
	Intervention	Control
Underweight^b^ (BMI < 18.5 kg/m^2^)		
Baseline	29/86 (34)	25/86 (29)
At 5 months	25/86 (29)	23/86 (27)
Normal^c^ (BMI ≥18.5 and < 25.0 kg/m^2^)		
Baseline	54/86 (63)	58/86 (67)
At 5 months	57/86 (66)	60/86 (70)
Overweight (BMI ≥25.0 and < 30.0 kg/m^2^)		
Baseline	3/86 (3)	3/86 (3)
At 5 months	4/86 (5)	3/86 (3)
Anemia^d, e^ (Hb < 12.0 g/dL)		
Baseline	19/85 (22)	23/85 (27)
At 5 months	19/85 (22)	22/86 (26)
Iron deficiency^f^ (FER^g^ < 15 μg/L)		
Baseline	15/84 (18)	21/84 (25)
At 5 months	17/85 (20)	13/86 (15)
Marginal iron stores^f^ (FER^g^ ≥ 15 and < 50 μg/L)		
Baseline	49/84 (58)	35/84 (42)
At 5 months	48/85 (56)	46/86 (54)
Tissue iron deficiency^f^ (sTfR > 8.3 mg/L)		
Baseline	7/84 (8)	10/84 (12)
At 5 months	5/85 (6)	11/86 (13)
Iron deficiency anemia^f^ (Hb < 12.0 g/dL and FER^g^ < 15 μg/L)		
Baseline	8/84 (10)	12/84 (14)
At 5 months	8/85 (9)	9/86 (10)
Vitamin A deficiency^f^ (RBP^g^ < 0.70 μmol/L)		
Baseline	0/84 (0)	0/84 (0)
At 5 months	0/85 (0)	0/86 (0)
Marginal vitamin A deficiency^f^ (RBP^g^ ≥ 0.70 and < 1.05 μmol/L)		
Baseline	7/84 (8)	3/84 (4)
At 5 months	8/85 (9)	7/86 (8)
Folate deficiency^h^ (< 3 ng/mL)		
Baseline	0/78 (0)	0/74 (0)
At 5 months	0/84 (0)	0/84 (0)
Marginal folate deficiency^h^ (≥3 and < 6 ng/mL)		
Baseline	21/78 (27)	24/74 (32)
At 5 months	10/84 (12)	18/84 (21)
Vitamin B12 deficiency^i^ (< 148 pmol/L)		
Baseline	0/83 (0)	1/84 (1)
At 5 months	NA	NA
Marginal vitamin B12 deficiency^i^ (≥148 and < 222 pmol/L)		
Baseline	2/83 (2)	5/84 (6)
At 5 months	NA	NA

*BMI* Body mass index, *Hb* Hemoglobin, *FER* Ferritin, *sTfR* Soluble transferrin receptor, *RBP* Retinol-binding protein, *NA* Not available

^a^Values are *n*/total *n* (%)

^b^Thereof mild underweight (BMI ≥17.0 and < 18.5 kg/m^2^): at baseline *n* = 21/*n* = 18 (intervention/control), at 5 months *n* = 19/*n* = 14; moderate underweight (BMI ≥16.0 and < 17.0 kg/m^2^): at baseline *n* = 6/*n* = 4, at 5 months *n* = 5/*n* = 6; severe underweight (BMI < 16.0 kg/m^2^): at baseline *n* = 2/*n* = 3, at 5 months *n* = 1/*n* = 3

^c^Thereof low-normal BMI (BMI ≥18.5 and < 20.0 kg/m^2^): at baseline *n* = 24/*n* = 24, at 5 months *n* = 21/*n* = 24

^d^At baseline total *n* = 170 (*n* = 1/*n* = 1 refused blood sampling). At 5 months total *n* = 171 (*n* = 1 intervention participant refused blood sampling)

^e^Thereof mild anemia (Hb ≥11.0 and < 12.0 g/dL): at baseline *n* = 13/*n* = 16, at 5 months *n* = 16/*n* = 14; moderate anemia (Hb ≥8.0 and < 11.0 g/dL): at baseline *n* = 6/*n* = 7, at 5 months *n* = 3/*n* = 8

^f^At baseline total *n* = 168 (*n* = 1/*n* = 1 refused blood sampling, *n* = 1/*n* = 1 with missing aliquot). At 5 months total *n* = 171 (*n* = 1 intervention participant refused blood sampling)

^g^Values adjusted for subclinical inflammation

^h^At baseline total *n* = 152 (*n* = 1/*n* = 1 refused blood sampling, *n* = 1/*n* = 1 with missing aliquot, *n* = 6/*n* = 10 with deviating duplicate measurement). At 5 months total *n* = 168 (*n* = 1 intervention participant refused blood sampling, *n* = 1/*n* = 2 with deviating duplicate measurement)

^i^At baseline total *n* = 167 (*n* = 1/*n* = 1 refused blood sampling, *n* = 2/*n* = 1 with missing aliquot). Values for vitamin B12 not available at 5 months

**Table 4:** Mean BMI, weight, TSF, and MUAMC at baseline and at 5 months (follow-up) by group and the intervention effects in female Cambodian garment workers^a^

	Group		Intervention effect		
	Intervention	Control	Mean difference	Cohen’s *d*	*p*
BMI, kg/m^2^					
Baseline	19.8 ± 2.4^b^	19.9 ± 2.4	–	–	–
At 5 months, unadjusted	19.9 ± 2.3	19.9 ± 2.4	–	–	–
At 5 months, adjusted	20.0 (19.8, 20.2)^c^	19.9 (19.7, 20.0)	0.1 (− 0.1, 0.4)	0.17	0.27
Weight, kg					
Baseline	46.0 ± 6.1	47.4 ± 6.3	–	–	–
At 5 months, unadjusted	46.4 ± 5.9	47.6 ± 5.9	–	–	–
At 5 months, adjusted	47.0 (46.7, 47.4)	46.9 (46.5, 47.3)	0.1 (−0.4, 0.7)	0.06	0.64
TSF, mm					
Baseline	15.4 ± 4.5	15.4 ± 4.8	–	–	–
At 5 months, unadjusted	15.6 ± 4.7	15.2 ± 4.6	–	–	–
At 5 months, adjusted	15.6 (15.1, 16.0)	15.2 (14.7, 15.7)	0.4 (−0.3, 1.1)	0.18	0.24
MUAMC, cm					
Baseline	19.1 ± 1.4	19.2 ± 1.6	–	–	–
At 5 months, unadjusted	19.1 ± 1.5	19.3 ± 1.5	–	–	–
At 5 months, adjusted	19.2 (19.0, 19.3)	19.3 (19.1, 19.4)	−0.1 (−0.3, 0.1)	−0.14	0.35

*BMI* Body mass index, *TSF* Triceps skinfold thickness, *MUAMC* Mid-upper arm muscle circumference

^a^Total *n* = 172 (completed the follow-up, *n* = 86 intervention and *n* = 86 control). A general linear model with adjustments for baseline values was used to predict marginal means (95% CIs) for each outcome variable and to estimate intervention effects as corresponding marginal mean differences (95% CIs) including an estimated standardized effect size (Cohen’s *d*)

^b^Mean ± SD (all such values)

^c^Marginal mean, 95% CI in parentheses (all such values)

**Table 5:** Mean Hb, FER, sTfR, RBP and folate concentrations at baseline and at 5 months (follow-up) by group and the intervention effects in female Cambodian garment workers^a^

	Group		Intervention effect		
	Intervention	Control	Mean difference	Cohen’s *d*	*p*
Hb, g/dL					
Baseline	12.6 ± 0.9 (85)^b^	12.4 ± 1.0 (85)	–	–	–
At 5 months, unadjusted	12.6 ± 0.9 (85)	12.3 ± 1.0 (86)	–	–	–
At 5 months, adjusted^c^	12.5 (12.4, 12.6) (85)^d^	12.4 (12.3, 12.5) (85)	0.1 (− 0.1, 0.3)	0.17	0.30
FER, μg/L^e^					
Baseline	40.4 ± 33.8 (84)	44.9 ± 40.0 (84)	–	–	–
At 5 months, unadjusted	38.0 ± 27.1 (85)	47.4 ± 39.6 (86)	–	–	–
At 5 months, adjusted^c^	39.3 (35.5, 43.0) (84)	45.8 (42.1, 49.6) (84)	−6.6 (−11.9, − 1.3)	− 0.39	0.015
sTfR, mg/L					
Baseline	5.8 ± 2.6 (84)	6.3 ± 3.3 (84)	–	–	–
At 5 months, unadjusted	5.9 ± 2.6 (85)	6.2 ± 3.5 (86)	–	–	–
At 5 months, adjusted^c^	6.1 (5.9, 6.3) (84)	5.9 (5.7, 6.1) (84)	0.2 (−0.1, 0.5)	0.23	0.15
RBP, μmol/L^e^					
Baseline	1.37 ± 0.26 (84)	1.49 ± 0.31 (84)	–	–	–
At 5 months, unadjusted	1.42 ± 0.33 (85)	1.44 ± 0.35 (86)	–	–	–
At 5 months, adjusted^c^	1.45 (1.39, 1.52) (84)	1.40 (1.34, 1.47) (84)	0.05 (−0.04, 0.14)	0.17	0.27
Folate, ng/mL					
Baseline	8.0 ± 3.1 (78)	7.8 ± 2.9 (74)	–	–	–
At 5 months, unadjusted	9.6 ± 4.5 (84)	8.2 ± 3.2 (84)	–	–	–
At 5 months, adjusted^c^	9.5 (8.8, 10.3) (78)	8.4 (7.6, 9.2) (73)	1.1 (−0.02, 2.2)	0.32	0.054

*Hb* Hemoglobin, *FER* Ferritin, *sTfR* Soluble transferrin receptor, *RBP* Retinol binding protein

^a^A general linear model with adjustments for baseline values was used to predict marginal means (95% CIs) for each outcome variable and to estimate intervention effects as corresponding marginal mean differences (95% CIs) including an estimated standardized effect size (Cohen’s *d*)

^b^Mean ± SD, *n* in parentheses (all such values)

^c^Among subjects with data for both time points

^d^Marginal mean, 95% CI and *n* in parentheses (all such values)

^e^Values adjusted for subclinical inflammation

### Intervention effects on anthropometric variables

The adjusted mean BMI at 5 months was 0.1 kg/m^2^ higher among the intervention group, representing a non-significant, very small to small effect (*p* = 0.27, Cohen’s *d* = 0.17). On the other hand, no considerable differences were observed between groups for adjusted mean weight. The adjusted mean TSF among the intervention group was higher by 0.4 mm, also illustrating a non-significant, very small to small effect (*p* = 0.24, Cohen’s *d* = 0.18). In contrast, adjusted mean MUAMC at 5 months was slightly lower, but not significantly, by 0.1 cm (*p* = 0.35, Cohen’s *d* = − 0.14) (Table 4). Unadjusted values generally did not deviate from results obtained by adjustment for baseline values (only the unadjusted impact on weight was slightly higher with + 0.2 kg).

**Fig. 2:**
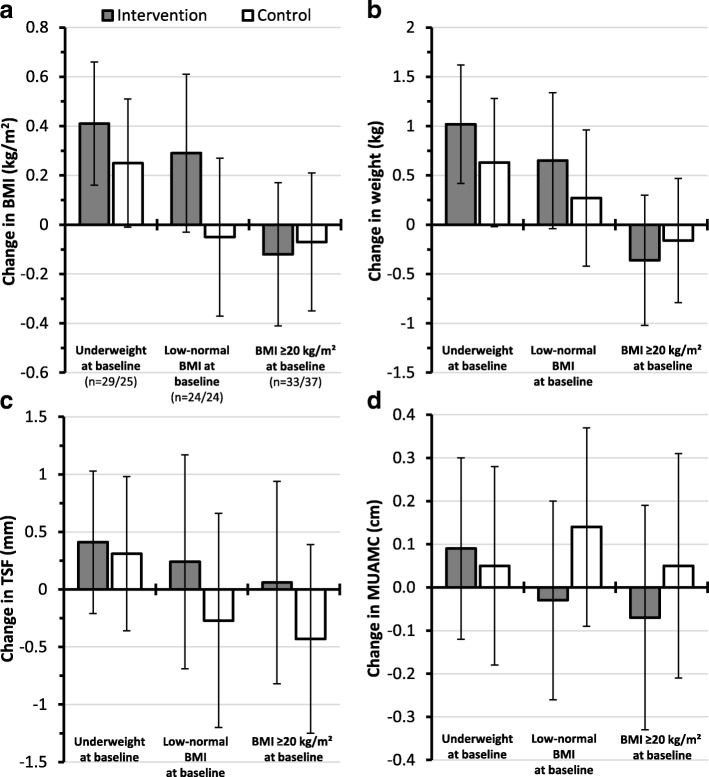
Mean change in **a** BMI, **b** weight, **c** TSF, and **d** MUAMC from baseline to 5 months (follow-up) by group and BMI status at baseline. Total *n* = 172 (completed the follow-up, *n* = 86 intervention and *n* = 86 control). Thereof underweight (BMI < 18.5 kg/m^2^) at baseline: *n* = 29/*n* = 25 (intervention/control); low-normal BMI (BMI ≥18.5 and < 20 kg/m^2^) at baseline: *n* = 24/*n* = 24; and BMI ≥20 kg/m^2^ at baseline: *n* = 33/*n* = 37. A general linear model with adjustments for baseline values was used to predict marginal mean changes (95% CIs) for each outcome variable. Whiskers illustrate corresponding 95% CIs. BMI: Body mass index; TSF: Triceps skinfold thickness; MUAMC: Mid-upper arm muscle circumference.

Subgroup analysis showed that adjusted means of BMI, weight, TSF, and MUAMC among underweight participants (BMI < 18.5 kg/m^2^) increased in intervention as well as in control subjects, with minor differences observed between groups only for BMI (around + 0.4 kg/m^2^ vs. + 0.2 kg/m^2^) and weight (about + 1.0 kg vs. + 0.6 kg). Differences were also found in participants with low-normal BMI at baseline (BMI ≥18.5 and < 20 kg/m^2^). Here, the adjusted mean BMI at follow-up was higher by around 0.35 kg/m^2^ in the intervention group (around + 0.3 kg/m^2^ vs. -0.05 kg/m^2^). Mean weight was likewise higher by approximately 0.4 kg (around + 0.7 kg vs. + 0.3 kg), as well as mean TSF by 0.5 mm (around + 0.2 vs. -0.3 mm). On the other hand, the adjusted mean MUAMC was slightly lower by around 0.2 cm in intervention participants. Furthermore, in workers with a BMI ≥20 kg/m^2^, mean BMI and mean weight marginally decreased in both groups, with no differences noticed. Adjusted mean TSF at 5 months was slightly higher by 0.5 mm (around + 0.1 mm vs. -0.4 mm), while mean MUAMC was marginally lower by approximately 0.1 cm in the intervention group (Fig. 2).

### Intervention effects on hemoglobin and micronutrient status

At the end of the intervention (Table 5), the adjusted mean Hb was 0.1 g/dL higher among the intervention group, a non-significant, very small to small effect (*p* = 0.30, Cohen’s *d* = 0.17). In contrast, mean FER was lower by 6.6 μg/L, illustrating a significant, small to medium negative effect (*p* = 0.015, Cohen’s *d* = − 0.39). Concurrently, mean sTfR was 0.2 mg/L higher, representing a non-significant, small negative effect (*p* = 0.15, Cohen’s *d* = 0.22). Apart from that, the adjusted mean RBP was 0.05 μmol/L higher among the intervention group, a non-significant, very small to small difference (*p* = 0.27, Cohen’s *d* = 0.17). At last, mean folate was higher by 1.1 ng/mL, outlining a non-significant, small to medium positive impact (*p* = 0.054, Cohen’s *d* = 0.32).

**Fig. 3:**
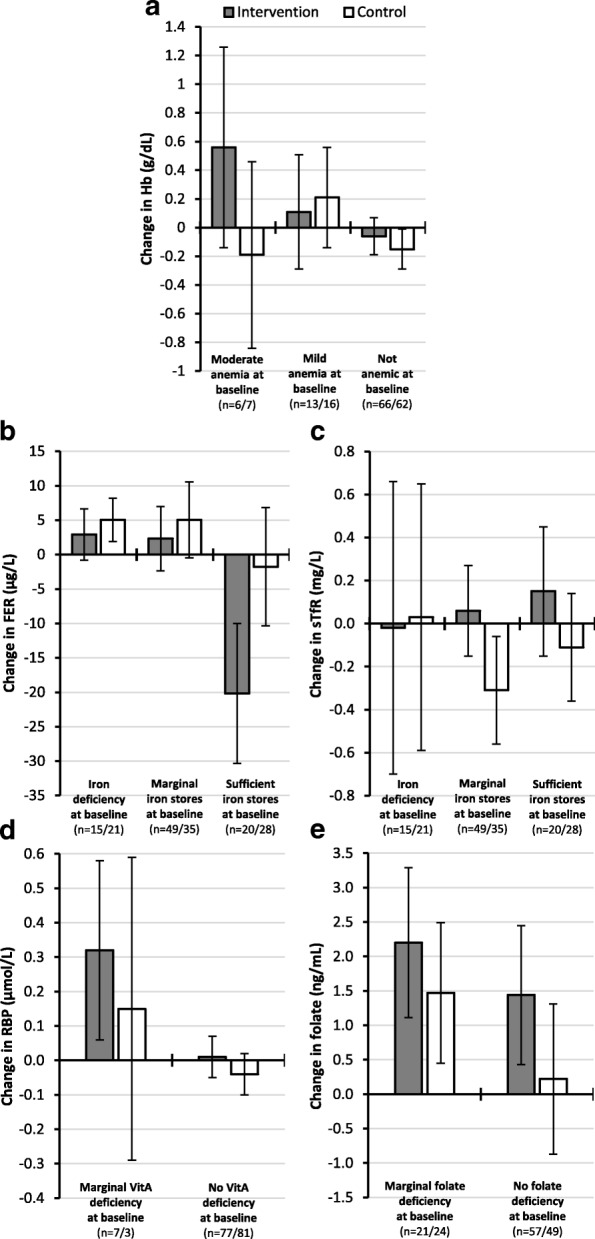
Mean change in **a** Hb, **b** FER, **c** sTfR, **d** RBP, and **e** folate concentrations from baseline to 5 months (follow-up) by group and status at baseline. A general linear model with adjustments for baseline values was used to predict marginal mean changes (95% CIs) for each outcome variable. Whiskers illustrate corresponding 95% CIs. **a** Mean change of Hb for subjects with moderate anemia (Hb ≥8.0 and < 11.0 g/dL), mild anemia (Hb ≥11.0 and < 12.0 g/dL), and no anemia (Hb ≥12.0 g/dL) at baseline. Total *n* = 85/*n* = 85 (intervention/control). **b** Mean change of FER for subjects with iron deficiency (FER < 15 μg/L), marginal iron stores (FER ≥15 and < 50 μg/L), and sufficient iron stores (FER ≥50 μg/L) at baseline. Total *n* = 84/*n* = 84. Values adjusted for subclinical inflammation. **c** Mean change of sTfR for subjects with iron deficiency, marginal iron stores, and sufficient iron stores at baseline. Total *n* = 84/*n* = 84. **d** Mean change of RBP for subjects with marginal VitA deficiency (RBP ≥0.70 and <1.05 μmol/L) and no VitA deficiency (RBP ≥1.05 μmol/L) at baseline. Total *n* = 84/*n* = 84. Values adjusted for subclinical inflammation. **e** Mean change of folate for subjects with marginal folate deficiency (folate ≥3 and < 6 ng/mL) and no folate deficiency (folate ≥6 ng/mL) at baseline. Total *n* = 78/*n* = 73. Hb: Hemoglobin; FER: Ferritin; sTfR: Soluble transferrin receptor; RBP: Retinol binding protein; VitA: Vitamin A.

In the secondary subgroup analysis (Fig. 3), mean change in Hb differed only among the few women with moderate anemia (Hb ≥8.0 and < 11.0 g/dL) at baseline. Here, the adjusted mean Hb at 5 months was higher by 0.8 g/dL in intervention participants (around + 0.6 g/dL vs. -0.2 g/dL). Overall, mean Hb slightly increased among the subjects with mild anemia (Hb ≥11.0 and < 12.0 g/dL), and marginally decreased for women not affected by anemia (Hb ≥12.0 g/dL). Mean FER slightly increased, for both groups, among workers affected by iron deficiency (FER < 15 μg/L), as well as among the subjects with marginal iron stores (FER ≥15 and < 50 μg/L). However, among women with marginal iron stores, sTfR was higher by 0.4 mg/L in intervention participants (around + 0.1 mg/L vs. -0.3 mg/L). On the other hand, mean change in FER clearly differed among groups in subjects with sufficient iron stores (FER ≥50 μg/L) at baseline. Here, mean FER at follow-up was lower by 18 μg/L in the intervention participants. In line with this finding, mean sTfR at 5 months was higher by 0.3 mg/L in intervention participants (around + 0.2 mg/L vs. -0.1 mg/L). The adjusted mean change in RBP differed only among the few women with marginal VitA deficiency (RBP ≥0.70 and < 1.05 μmol/L) at baseline. Mean RBP at follow-up was higher by approximately 0.2 μmol/L in intervention participants. Mean folate considerably increased, for both groups, among workers affected by marginal folate deficiency (folate ≥3 and < 6 ng/mL), and was higher by 0.7 ng/mL in intervention participants (around + 2.2 ng/mL vs. + 1.5 ng/mL). Mean change also clearly differed between groups in subjects not affected by folate deficiency (folate ≥6 ng/mL), where folate at 5 months was higher by 1.2 ng/mL for intervention participants (+ 1.4 ng/mL compared with + 0.2 ng/mL).

## Discussion

This model lunch provision for 5 months resulted in marginally increased mean BMI, mean weight, mean TSF, and a nominal lower mean MUAMC. These insignificant results, illustrating negligible to very small/small effects, are assumed to represent, if any, a very limited general impact on worker’s anthropometry. But, subgroup analysis suggests a more pronounced intervention impact on weight (around + 0.4 kg) and BMI (up to + 0.35 kg/m^2^) among underweight participants and those with a low-normal BMI. Furthermore, in subjects with low-normal BMI and those with a BMI ≥20 kg/m^2^, TSF was thicker by 0.5 mm. Although not being suited to test for statistical significance, such effects would actually correspond to small to medium effects (Cohen’s *d* between ≥0.2 and < 0.5).

Food provision trials in low-income countries primarily focus on school feeding programs. Interestingly, evidence of the impact on anthropometric indices remains inconclusive [39]. In Cambodia, the recent ILO multi-factory study reported that 1 year of daily food provision did not induce changes of the mean BMI of Cambodian garment workers [9]. However, food provision within the ILO study differed substantially in its intervention factories (ranging from snacks to full lunches). Therefore, comparisons are difficult to draw. Moreover, ILO-study participants were distinctly older and to a lesser extent affected by underweight than workers in the present study.

Effects from lunch provision on worker’s anthropometric indices might have been weakened due to the frequent onset of infectious diseases, as infections are known to have a negative impact on the nutritional status [40]. At baseline, participants often reported symptoms of respiratory tract infections, fever, and diarrhea, and tended to continue work despite being sick [4].

The study collected qualitative data on the dietary intake through 24 h-recalls among all participants at several interviews during the intervention [19]. Results indicate that some participants tended to skip breakfasts, but hardly ever skipped lunch meals. Therefore, providing lunch to workers replaces meals that are otherwise eaten by the women, mostly low-price options from nearby street vendors and/or home prepared food items. Consequently, total dietary surplus (e.g. of calories, one prerequisite to expect effects on anthropometry) through lunch provision might be restricted. Although skipping of breakfasts somewhat increased in intervention subjects, no significant differences in skipping breakfasts between groups were observed [19]. Yet, skipping of meals in workers with access to a staff canteen should be closely monitored. Moreover, a distinct lower consumption of energy-dense sweets/sugared beverages was noted in intervention participants [19]. This effect is regarded as beneficial for the prevention of non-communicable chronic diseases although it also lowers total energy intake [41].

Overall, lunches matched recommendations on the energy content of lunch provision through canteens [42], as well as recommendations for sources of food energy [43]. Nevertheless, the RDA of 2115 kcal/day [20] might underestimate energy requirements among workers, notably in those with a BMI < 20 kg/m^2^ and those exposed to heavy work load. Consequently, an adjusted higher amount of calories during lunch provision might be needed to achieve a stronger effect on the BMI of laborers with suboptimal nutritional status. On the other hand, any lunch provision program should also consider the presence of normal weight and overweight workers.

The model lunch sets had a low mean iron content [18]. Due to their relatively high price, animal source foods were served in small portion sizes of ~ 50 g/day, equaling 0.5–1.7 mg iron per 100 g edible portion [18, 44–46]. Consequently, most of the dietary iron was provided as less bioavailable nonheme iron in vegetables, fruits, and rice [18]. As vitamin C enhances nonheme iron absorption, sets provided on average a relatively high amount of vitamin C. But, the effect might have been limited in a complete menu containing various components known to inhibit iron intake [47, 48]. Although data on the dietary iron intake among Cambodian garment workers are missing, the lunch sets could have contained less iron than lunches eaten by the workers outside the factory gates. Alternative and affordable heme iron-rich food items (e.g. blood curd, liver, or certain small fish species) could constitute a suitable option to increase the iron content [18, 49–51].

If the obtained overall finding in terms of Hb represents an intervention effect can be questioned. The unadjusted mean Hb remained unchanged in intervention participants. However, three out of four study subjects were not anemic at baseline. Therefore, distinct positive effects on mean Hb concentration could not be expected from the intervention among non-anemic participants. On the other hand, the observed changes in mean Hb among subjects affected by moderate anemia are considered relevant. The prevalence of anemia was initially expected to be higher, since data indicate that 45% of Cambodian women of reproductive age are anemic [10]. The recent ILO survey reported a similar high prevalence in female garment workers [9]. Yet, these findings are based on capillary blood analysis. In a recent study among children from Laos, Hb concentration by HemoCue was significantly higher in venous blood samples compared to capillary blood, resulting in different anemia prevalence data [52]. Although Hb measurement via HemoCue is thought to be more reliable in venous samples [53], some report that HemoCue showed poor agreement compared with automated hematology analyzers [52].

Iron deficiency is believed to be the primary cause of anemia [11]. However, as previously mentioned, the iron content of the studied lunch sets was relatively low [18]. On the other hand, iron deficiency can only partially explain anemia in this study population [4], as the prevalence of iron deficiency anemia among subjects was solely 12%. Strategies to improve zinc and folate status, as well as to treat and prevent hookworm infections, have been suggested [54]. In addition, genetic disorders, e.g. Hb E variants and α-thalassemia, are reported to affect > 50% of the Cambodian population, causing lower Hb concentrations regardless of iron stores [16, 54–57]. In a recent one-year randomized controlled trial, neither iron ingots added to cooking pots nor daily iron supplements (18 mg/d) increased Hb concentration in anemic Cambodian women [58]. In comparison, daily high-dose iron supplementation (60 mg/d) for 12 weeks increased Hb in a female study population in Cambodia, while added multiple micronutrients did not confer additional benefits [56].

None of the study subjects were affected by frank VitA deficiency and only few participants showed a marginal VitA status at baseline, which is in line with recent national representative data for women of reproductive age [34, 54]. The uptake of VitA from the diet is under homeostatic control [59], consequently, no effects on RBP concentrations could be expected in VitA-replete subjects. The overall trend on increasing RBP, is largely based on the increase of mean RBP in few intervention subjects with marginal VitA deficiency, which is expected to be relevant, but confirmation is needed in a larger study including more participants with suboptimal VitA status. At the time of planning, the study population was expected to be more affected by a poor VitA status, given foregoing findings [60].

The results suggest that lunch sets provided a beneficial amount of dietary folate. The estimated mean folate content among sets was corresponding to 44% of the SEA-RDA [20]. In addition, missing folate data in local food composition tables certainly led to an underestimation for some lunch sets [18]. The finding on the prevalence of marginal folate deficiency among workers is in line with previous reports that suggest measures to increase folate/folic acid intake of Cambodian women [54]. According to the subgroup analysis, the trend on folate status not only concerned participants with marginal folate deficiency, but also subjects with adequate folate status. However, a part of the increase in folate concentration among intervention participants with marginal folate status can be attributed to the upregulation of folate uptake from the diet [61], as represented by the increase in mean folate in control participants with a marginal folate status.

### Limitations of the study

Results of this study are related to the setting and the specific study population. However, the status of the laborers and the working conditions were assumed to be comparable with general conditions in the Cambodian garment industry. Moreover, the study’s inclusion criteria represented a greater part of workers employed by this sector.

The model lunch sets could not be based on the study’s baseline findings [4] nor on any other previous gap-oriented assessment. An appropriate intervention duration, as well as proper amounts of calories or micronutrients, to specifically target underweight, anemia and/or micronutrient deficiencies, could not be established beforehand. Furthermore, the estimation of the lunches’ nutritive value was limited [18]. For instance, no information was available about components known to inhibit iron bioavailability.

Fear and skepticism related to the blood sampling were reported by workers, notably due to a severe HIV outbreak caused by clinicians reusing syringes shortly before enrollment [62]. Moreover, the factory unexpectedly changed its main purchaser and a part of its management at the time when the study started. As a consequence, a part of the total factory staff, and therefore also a relatively high number of workers who had already signed consents or were already enrolled, ceased the work and left the factory between April and June 2015. Almost all study dropouts fell in this period. Yet, they were equally distributed across groups and their sociodemographic characteristics were comparable to those who completed the study (data not shown). Given the high fluctuation rate, the impact assessment was performed after 5 months already. As the number of data sets was smaller than targeted, this clearly limited the statistical power and the effect sizes that could be measured. In line with guidelines for exploratory studies, no corrections for multiple comparisons have been made [63].

Only a part of the enrolled workers where actually affected by underweight, anemia and/or micronutrient deficiencies. However, direct improvements can only be expected in malnourished individuals. The subgroup analysis was based on relatively small sample sizes, holding a descriptive character, and indicating trends only. The calculation of the sample size of future studies may consider the initial prevalence of malnutrition.

The prevalence of hemoglobinopathies, which are likely to be a contributing factor to anemia, was not measured. Inherited hemoglobin disorders are also known to impact on markers of iron status [16, 64]. Furthermore, menstrual blood loss, a determinant of iron stores in women of reproductive age [65], could not be recorded.

## Conclusions

After 5 months of lunch provision, anthropometric variables merely showed non-significant marginal distinctions between the intervention and the control group. Yet, subgroup analysis prompts that effects differ according to the initial status of workers. For instance, the positive impact on BMI and weight was found more pronounced in women with a poor or marginal nutritional status. However, given the low sample size in subgroups, trends, but no definite inferences can be stated. Overall, only minor non-significant positive differences were noticed in Hb and VitA status for the intervention participants. Specific results indicate that the model lunch sets need to be revisited for iron content and/or iron bioavailability, as interventions subjects showed significantly lower FER values at the follow-up. On the other hand, endline folate status was higher in workers with access to the lunch provision, although insignificantly, advising that lunch sets provided a relevant amount of dietary folate. In conclusion, lunch provision through a canteen for Cambodian garment workers is feasible and is believed to have the potential to result in positive effects on anthropometry, Hb, and micronutrient status, particularly in malnourished individuals. The authors suggest that similar trials with larger study populations, which include lunch sets adapted to identified requirements of workers affected by underweight, anemia and/or definite micronutrient deficiencies, should be performed. The overall findings from this study should have practical implications for the design and implementation of subsequent studies, lunch programs, and further strategies aiming at the improvement of the nutritional situation of female garment workers in Cambodia.

## Data Availability

The datasets generated and analyzed during the study are not publicly available due the terms of consent to which the participants agreed but are available from the corresponding author on reasonable request.

## Abbreviations

AGP: α1-acid-glycoprotein
AI: Adequate intake
BMI: Body mass index
CDC: Centers for Disease Control and Prevention
CI: Confidence interval
CRP: C-reactive protein
FER: Ferritin
Hb: Hemoglobin
HIV: Human immunodeficiency virus
ILO: International Labour Organization
Kcal: Kilocalories
LUPROGAR: Lunch provision in garment factories
Max.: Maximum
Min.: Minimum
MUAC: Mid upper-arm circumference
MUAMC: Mid upper-arm muscle circumference
NGO: Non-government organization
RAE: Retinol activity equivalent
RBP: Retinol binding protein
RDA: Recommended dietary allowance
SD: Standard deviation
SEA: Southeast Asia
sTfR: Soluble transferrin receptor
TSF: Triceps skinfold thickness
USD: United States Dollar
VitA: Vitamin A
VitB12: Vitamin B12
